# Willingness to pay for supplementary medical insurance and its influencing factors among rural residents in ethnic minority areas

**DOI:** 10.3389/fpubh.2026.1733751

**Published:** 2026-03-04

**Authors:** Yao Li, Daiqing Cao, Mingwei Zhu, Lili Zhu, Liang Shen, Xiang He

**Affiliations:** 1 School of Management, Xuzhou Medical University, Xuzhou, Jiangsu, China; 2 The First People’s Hospital of Yancheng, Yancheng Clinical College of Xuzhou Medical University, Yancheng, Jiangsu, China; 3 Medical Affairs Department, Affiliated Hospital of Xuzhou Medical University, Xuzhou, Jiangsu, China

**Keywords:** Andersen model, influencing factors, rural areas, supplementary medical insurance, willingness to pay

## Abstract

**Background:**

In recent years, the prices of medical services have been constantly rising, and supplementary medical insurance has become an important way for some residents to alleviate their heavy economic burden. Due to their special natural conditions and social environment, residents in rural areas of ethnic minorities face higher risks and costs in seeking medical treatment. Therefore, they have personalized demands for supplementary medical insurance. Therefore, studying the willingness of residents in rural areas of ethnic minorities to pay supplementary medical insurance and its influencing factors has important practical and policy significance.

**Methods:**

Data Collection: A questionnaire was designed based on the Andersen model, and a mixed sampling approach was employed to conduct offline surveys in the market areas of five towns in Tongdao County; concurrently, electronic questionnaires were distributed via social media applications. A total of 551 valid questionnaires were obtained. Data Analysis: Data were analyzed using SPSS. Descriptive statistics, hierarchical logistic regression, and multiple correspondence analysis were applied to examine how different factors influence willingness to pay among rural residents in ethnic minority areas.

**Results:**

58.98% of rural residents were willing to pay for supplementary medical insurance. Multivariate analysis indicated that occupation, emphasis on health management, annual household income, payment experience, and family medical expenses in the past year significantly influenced willingness to pay (*p* < 0.05). Within the Andersen model framework, enabling resources exerted the strongest impact, whereas demand factors had the weakest. Furthermore, multiple correspondence analysis indicated that residents with payment experience, an annual household income between 100,000 and 150,000 yuan, and those employed in party, government, or public institutions were more willing to pay for supplementary medical insurance.

**Conclusion:**

All three categories of factors within the Andersen model framework significantly affected rural residents’ willingness to pay in Tongdao County. A disconnect between willingness to pay and actual behavior concerning supplementary medical insurance was observed in the rural areas of Tongdao County. The construction and improvement of supplementary medical insurance in this context require joint efforts from all stakeholders to develop sustainably.

## 1 Introduction

Healthcare is essential to safeguarding people’s basic living standards and meeting their growing demand for a better quality of life. The report to the 20th National Congress of the Communist Party of China noted that China still has structural shortcomings in areas such as healthcare and older population care, and emphasized the need to accelerate the improvement of the social security system, including medical and older population care coverage, while promoting the orderly linkage and coordinated development of a multilevel medical security system (1). The 2025 Government Work Report further emphasized the need to continuously improve the capacity of healthcare services, with a particular focus on strengthening the development of medical resources and services at the primary level (2). These policy directions indicate that building a medical security system with broader coverage and clearer tiers, especially by improving supplementary medical insurance arrangements beyond basic medical insurance, has become a key priority in deepening the reform of China’s medical security system. With ongoing economic development, residents in ethnic minority areas are increasingly seeking more varied health insurance options. Meanwhile, factors such as the natural environment, transportation conditions, and local customs create greater uncertainty and risk related to access to healthcare services, exposure to region-specific diseases, and the resulting financial burden (3). In this context, supplementary medical insurance has become an important policy tool for improving the medical security system, easing the financial burden of healthcare, and meeting residents’ individualized insurance needs. Its institutional fit in rural ethnic minority areas, as well as residents’ awareness, acceptance, and willingness to participate, needs to be systematically assessed (4).

Supplementary medical insurance refers to voluntary forms of coverage beyond the scope of basic medical insurance, provided by enterprises and social organizations with state support to enhance individuals’ levels of medical security. It encompasses various forms of insurance, including critical illness insurance, commercial health insurance, enterprise supplementary medical insurance, and medical subsidies for civil servants (5). In recent years, research on supplementary health insurance has gradually increased alongside economic development and the diversification of residents’ medical insurance needs. Examples include studies on the impact of critical illness insurance on fraud victims (6), the effects of supplementary private health insurance on reducing households’ poverty vulnerability and residents’ medical expenditures (7, 8), and research on the impact of supplementary health insurance on out-of-pocket expenses for older population households in urban areas and on payments for community hospital services (9, 10). Overall, existing studies have largely focused on multilevel medical security systems in urban or more developed areas, with particular attention to payment patterns for supplementary medical insurance and their impacts. In contrast, rural ethnic minority areas and other less developed regions have received limited attention, and the mechanisms shaping residents’ willingness to pay for supplementary medical insurance, along with the associated influencing factors, remain insufficiently examined in a systematic and in depth way.

Willingness to pay (WTP) refers to an individual’s considered psychological inclination to participate in or purchase a certain type of insurance. A positive WTP promotes the tendency to purchase insurance, whereas a negative WTP reduces the intention to do so (11). In this study, WTP denotes rural residents’ proactive inclination to pay for supplementary medical insurance and their attitudes toward this behavior. Most existing studies have focused on the payment status and subsequent effects of supplementary medical insurance. At present, studies on the determinants of willingness to pay have examined factors such as supply side incentives, the design of payment mechanisms, and the coverage of basic medical insurance (12–14). Other studies have investigated how individual, social, and household level factors, including household debt, healthcare utilization and medical expenditure, and individual health status, influence willingness to pay for supplementary medical insurance and the pathways through which these effects occur (15–17). However, these studies generally have two limitations. First, they mostly adopt a system or supply side perspective and rarely conduct detailed research from the demand side, especially on individuals’ psychological perceptions and cognition. Second, they tend to examine factors in isolation and lack a structured analysis grounded in a systematic behavioral model, which makes it difficult to fully capture the complex mechanisms through which willingness to pay is formed.

The Andersen model is a research framework used to explain and analyze the behavior and patterns of health service utilization. It is currently widely applied in research on medical and health services and related derived demands, and is also suitable for analyzing the influencing factors of health security behaviors and related service utilization (18). However, when applying the Andersen model to examine willingness to pay for supplementary medical insurance, existing studies rarely incorporate internal psychological mechanisms such as residents’ risk perception and value perception. Risk perception refers to an individual’s subjective assessment of the characteristics and severity of a specific risk. Residents’ perceptions of health risks, such as whether they routinely pay attention to health management, can affect their willingness to pay (11). Perceived value is a consumer’s subjective evaluation after weighing the benefits and costs of a product or service. When residents perceive higher value, for example, when they believe the premium is lower than the expected reimbursement, their willingness to pay tends to be higher (19). Therefore, this study builds on the Andersen model and draws on theoretical perspectives such as risk perception and perceived value to systematically examine rural residents’ willingness to pay for supplementary medical insurance in ethnic minority areas and its influencing factors.

In summary, this study takes residents in ethnic minority rural areas as the study population. Based on the Andersen model and incorporating residents’ perceptions of health risks and their perceived value of supplementary medical insurance, a questionnaire was designed covering residents’ socioeconomic characteristics, health status, insurance awareness, and risk attitudes. A field sampling survey was conducted. Statistical methods were employed to systematically analyze the factors influencing WTP among residents in rural ethnic minority areas and the reasons underlying their pathways of influence. On this basis, targeted suggestions and countermeasures are proposed, along with policy recommendations to facilitate the transformation of enrollment intention into effective enrollment behavior, providing theoretical support and an empirical basis for advancing improvements to the multi-level medical security system in rural areas and for policy optimization.

## 2 Materials and methods

### 2.1 Data source

The study subjects were residents from rural areas of Tongdao Dong Autonomous County, Huaihua City, Hunan Province. The inclusion criteria for respondents were as follows: age 16 years or older; a certain educational foundation (with knowledge of basic medical insurance); and voluntary participation in the survey. Efforts were made to ensure that the sample encompassed residents with diverse demographic characteristics. This study collected data using a mixed sampling approach that combined offline intercept surveys with online self administered questionnaires. In January 2024 and March 2025, trained interviewers conducted offline intercept surveys in township market areas. Five townships, including Boyang Town and Xianxi Town, were randomly selected. In each township, about 80 to 90 residents completed the questionnaire on site, either by self completion or through interviewer administered questions. A total of 434 questionnaires were collected. After removing questionnaires with substantial missing information or obvious logical inconsistencies, 425 valid paper questionnaires were retained. For the online survey, an electronic questionnaire link was distributed via social media. IP restrictions were applied, and respondents could submit the questionnaire only after completing all items and spending at least 30 s, to reduce duplicate and invalid responses. A total of 150 questionnaires were distributed, and 136 were returned. After excluding invalid responses, 126 valid online questionnaires remained. In total, 551 valid questionnaires were obtained, yielding an effective response rate of 94.34%. Prior to the survey, investigators informed participants of the purpose of the questionnaire and the confidentiality measures. Questionnaires were administered only after obtaining consent. After collection, the data were entered into Excel, followed by data cleaning and logical error correction to establish a raw database.

### 2.2 Research model and questionnaire design

**Figure 1:**
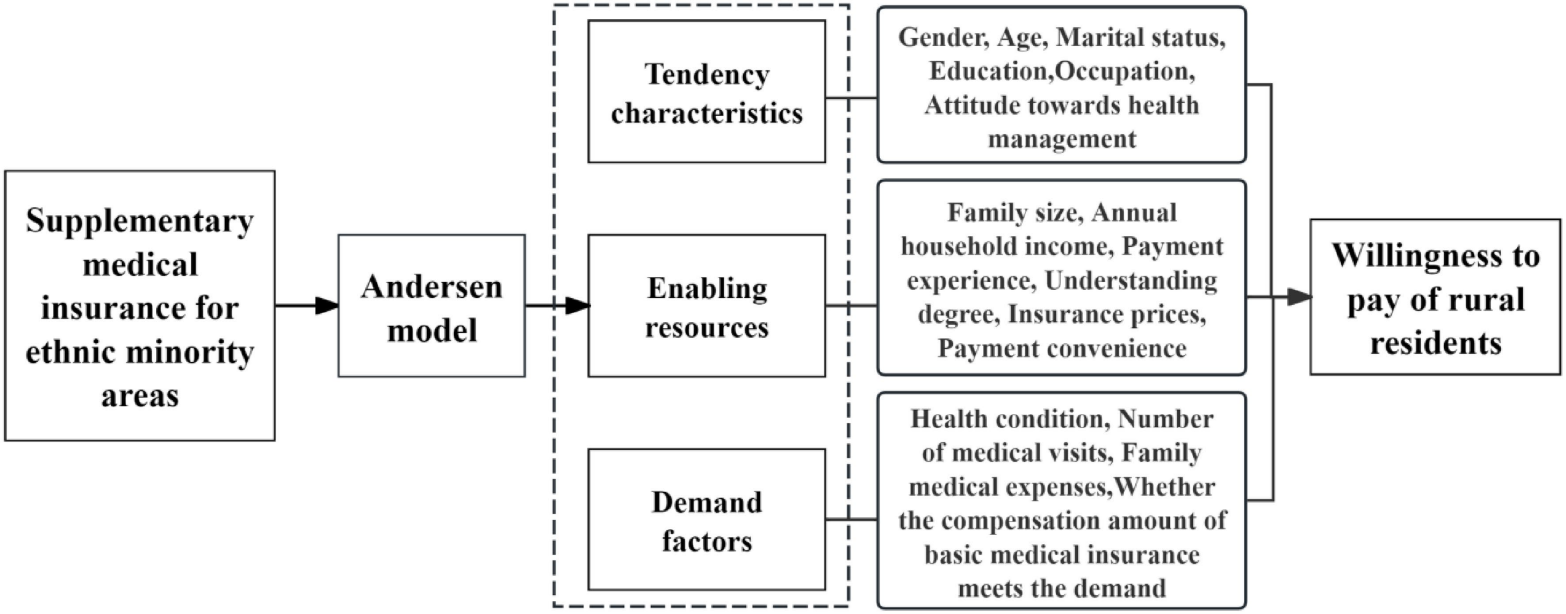
A supplementary medical insurance research framework based on the Andersen model.

This study uses the Andersen model as the primary theoretical framework and systematically analyzes rural residents’ willingness to pay for supplementary medical insurance and its influencing factors from three dimensions: tendency characteristics, enabling resources, and demand factors. Building on the Andersen model, the questionnaire design also draws on risk perception theory and perceived value theory. Because residents’ attitudes toward health risk and their perceptions of the value of supplementary medical insurance may influence willingness to pay, corresponding factors were added. Based on this, we developed a research model of the influencing factors of willingness to pay for supplementary medical insurance, as shown in Figure 1. The specific variables included in this study and their coding are presented in Table 1.

**Table 1:** Variables and assignments.

Variables			Assignments
Dependent variable		Willingness to pay	No = 0; Yes = 1
Independent variables	Tendency characteristics	Gender	Male = 0; Female = 1
		Age(year)	16–30 = 1; 31–40 = 2; 41–50 = 3; 51–60 = 4; >60 = 5
		Marital status	Unmarried = 0; Married = 1
		Education	Primary school and below = 1; Middle school = 2; High/secondary vocational school = 3; Junior college = 4; Bachelor’s degree and above = 5
		Occupation	Party and government employee = 1; Company employee = 2; Farmer = 3; Flexible employment personnel = 4; Other = 5
		Emphasizing health management	No = 0; Yes = 1
	Enabling resources	Family size(people)	1–2 = 1; 3–4 = 2; 5–6 = 3; 7–8 = 4; ≥9 = 5
		Annual household income(yuan)	≤60,000 = 1; 60,000–100,000 = 2; 100,000–150,000 = 3; 150,000–200,000 = 4; >200,000 = 5
		The degree of understanding of supplementary medical insurance	Very unfamiliar = 1; Slight unfamiliar = 2; Moderately familiar = 3; Quite familiar = 4; Very familiar = 5
		Has paid supplementary medical insurance	No = 0; Yes = 1
		The degree of influence of insurance prices	Very impact = 1; Quite impact = 2; Moderate impact = 3; Slight impact = 4; No impact = 5
		The degree of influence of payment convenience	Very impact = 1; Quite impact = 2; Moderate impact = 3; Slight impact = 4; No impact = 5
	Demand factors	Health condition in the past year	Very good = 1; Better = 2; Generally = 3; Poor = 4; Very poor = 5
		The number of medical visits in the past year(times)	0 = 1; 1–3 = 2; 4–7 = 3; 8–12 = 4; ≥13 = 5
		Family medical expenses in the past year	≤3,000 = 1; 3,000–8,000 = 2; 8,000–15,000 = 3; 15,000–30,000 = 4; >30,000 = 5
		The compensation of basic medical insurance meets the demand	No = 0; Yes = 1

Based on the aforementioned research model, the questionnaire is divided into two parts. The first part is the preface, which explains the purpose and significance of the survey, the research methods, and the concept of supplementary medical insurance. The second part is the main questionnaire, which covers items based on the research model and includes measurements of residents’ demographic and socioeconomic characteristics, health status, insurance awareness, risk perception, perceived value, and willingness to pay. The second part is the main body. It begins by collecting residents’ information through questions and answers structured around the three core factors of the Andersen model: Tendency characteristics: such as gender (20), age (21), marital status (18), education (22), occupation (23), and attitude toward health management (24). Enabling resources: such as family size, annual household income (25), prior experience with paying for supplementary medical insurance (20), the degree of understanding of it (19), insurance prices (25), and the degree of influence of payment convenience influences willingness to pay (13, 26). Demand factors: such as health condition in the past year (17, 27), number of medical visits in the past year (21), family medical expenses in the past year (16), and whether the compensation of basic medical insurance meets the demand (14). Subsequently, the questionnaire investigates residents’ willingness to pay (WTP), specifically whether they are willing to pay for supplementary medical insurance. Finally, respondents are thanked again for their participation. To ensure the accuracy of the questionnaire results, an attention-check question was included to identify and exclude invalid responses.

### 2.3 Statistical methods

The survey data were organized using Excel and analyzed with SPSS 26.0. First, descriptive statistics for categorical variables were reported as frequencies. Chi-square tests were then used to conduct a preliminary descriptive analysis of the associations between willingness to pay and each independent variable, to explore the patterns of relationships among variables. Then, based on the Andersen model, hierarchical binary logistic regression models were constructed to examine the factors associated with rural residents’ willingness to pay for supplementary medical insurance. Given that data were collected at two time points, in 2024 and 2025, a dummy variable for survey wave was included in all regression models to control for potential time effects. Finally, multiple correspondence analysis was conducted as a supplementary analysis to examine the relationships between willingness to pay and key influencing factors. Statistical significance was set at *p* < 0.05.

## 3 Results

### 3.1 Descriptive analysis of rural residents’ willingness to pay

Among the 551 rural residents, females accounted for 51.36%, indicating a roughly balanced gender distribution. Residents aged 16–60 constituted 93.1% of the sample, and 60.43% were married, as shown in Table 2. According to the survey data, 325 residents in the rural areas of Tongdao Dong Autonomous County were willing to pay for supplementary medical insurance, accounting for 58.98%. Specifically, 61.94% of male residents were willing to pay, compared with 56.18% of female residents. Among different age groups, residents aged 41–50 exhibited the highest willingness to pay, at approximately 67%. The proportion of unmarried residents willing to pay was higher than that of married residents. Furthermore, residents with higher education levels demonstrated a greater willingness to pay, with 79.46% of those holding a bachelor’s degree or above expressing willingness. In terms of occupation, residents employed in Party, government, or public institutions and those in enterprises showed the highest willingness to pay, at 92.40% and 63.93%, respectively.

**Table 2:** Descriptive statistics of the willingness to pay of rural residents.

Variables	*N*	Willingness to pay		$\chi^{2}$	*P*
		No	Yes		
Tendency characteristics					
Gender				1.885	0.17
Male	268	102	166		
Female	283	124	159		
Age (year)				7.553	0.109
16–30	177	65	112		
31–40	155	73	82		
41–50	97	32	65		
51–60	84	38	46		
>60	38	18	20		
Marital status				0.612	0.434
Unmarried	218	85	133		
Married	333	141	192		
Education				31.388	<0.001
Primary and below	89	45	44		
Middle	118	64	54		
High/secondary	111	44	67		
Junior	121	50	71		
Bachelor and above	112	23	89		
Occupation				55.034	<0.001
Government	79	6	73		
Company	102	44	78		
Farmer	126	71	55		
Flexible	136	65	71		
Other	108	50	58		
Emphasizing health management				15.588	<0.001
No	175	93	82		
Yes	376	133	243		
Enabling resources					
Family size (people)				2.973	0.562
1–2	49	18	31		
3–4	260	104	156		
5–6	182	79	103		
7~8	42	15	27		
≥9	18	10	8		
Annual household income(yuan)				45.893	<0.001
≤60,000	141	86	55		
60,000–100,000	248	101	147		
100,000–150,000	123	25	98		
150,000–200,000	25	8	17		
>200,000	14	6	8		
The degree of understanding of supplementary medical insurance				4.562	0.335
Very unfamiliar	64	30	34		
Slight unfamiliar	151	68	83		
Moderately familiar	174	70	104		
Quite familiar	113	43	70		
Very familiar	49	15	34		
Has paid supplementary medical insurance				115.95	<0.001
No	280	177	103		
Yes	271	49	222		
The degree of influence of insurance prices				20.261	<0.001
Very impact	98	48	50		
Quite impact	124	59	69		
Moderate impact	184	82	102		
Slight impact	94	25	69		
No impact	51	12	39		
The degree of influence of payment convenience				10.978	0.027
Very impact	44	11	33		
Quite impact	146	65	81		
Moderate impact	191	89	102		
Slight impact	119	46	73		
No impact	51	15	36		
Demand factors					
Health condition in the past year				6.457	0.167
Very good	149	60	89		
Better	284	125	159		
Generally	82	33	49		
Poor	30	7	23		
Very poor	6	1	5		
The number of medical visits in the past year(times)				6.066	0.194
0	175	71	104		
1–3	259	116	143		
4–7	87	32	55		
8–12	21	5	16		
≥13	9	2	7		
Family medical expenses in the past year				10.094	0.039
≤3,000	188	78	110		
3,000–8,000	214	96	118		
8,000–15,000	91	39	52		
15,000–30,000	36	7	29		
>30,000	22	6	16		
The compensation of basic medical insurance meets the demand				6.171	0.013
No	212	73	139		
Yes	339	153	186		

The preliminary analysis of willingness to pay and potential influencing factors showed that, among tendency characteristics, education level, occupation, and attention to health management were significantly associated with willingness to pay (*p* < 0.05), whereas sex, age, and marital status were not (*p* > 0.05). Among enabling resources, family size and understanding of supplementary medical insurance were not significantly associated with willingness to pay, while annual household income, prior purchase experience, insurance prices, and purchasing convenience showed significant associations, with prior purchase experience showing the strongest association. Among demand factors, family medical expenses in the past year and whether the compensation of basic medical insurance met the demand were significantly associated with willingness to pay, whereas self rated health status and the number of healthcare visits in the past year were not.

### 3.2 Logistic regression analysis of rural residents’ willingness to pay

To systematically examine the factors influencing willingness to pay for supplementary medical insurance among rural residents in ethnic minority areas, we conducted multivariable analyses based on the Andersen model using binary logistic regression. Willingness to pay for supplementary medical insurance was treated as the dependent variable (willing = 1, not willing = 0). Regression models were built in a stepwise manner across three blocks: tendency characteristics, enabling resources, and demand factors. Model 1 included only tendency characteristics. Model 2 added enabling resources to Model 1. Model 3 further incorporated demand factors, allowing a comprehensive assessment of how factors at different levels influence willingness to pay. In addition, because the survey data were collected at two different time points, a survey wave variable was included in all models. The regression results are presented in Table 3.

**Table 3:** Logistic regression results of the influencing factors of willingness to pay based on the Andersen model.

Variables	Model 1			Model 2			Model 3		
	*b*	*P*	*OR*	*b*	*P*	*OR*	*b*	*P*	*OR*
Survey period (Reference: 2024.01)									
2025.03	−0.001	0.995	0.999	0.141	0.589	1.152	0.207	0.471	1.230
Tendency characteristics									
*Gender (Reference: Male)*									
Female	−0.198	0.302	0.820	0.056	0.806	1.058	0.083	0.738	1.087
*Age (Reference: 16-30)*									
31–40	−0.457	0.093	0.633	−0.633	0.051	0.531	−0.586	0.078	0.557
41–50	0.501	0.125	1.650	0.349	0.375	1.418	0.422	0.314	1.524
51–60	0.041	0.909	1.041	−0.026	0.952	0.975	0.049	0.913	1.051
>60	0.104	0.809	1.110	−0.680	0.226	0.507	−1.094	0.077	0.335
*Marital status (Reference: unmarried)*									
Married	−0.132	0.561	0.877	0.012	0.964	1.012	0.031	0.914	1.031
*Education (Reference: primary and below)*									
Middle	−0.213	0.499	0.808	−0.174	0.649	0.841	−0.057	0.888	0.945
High/secondary	0.316	0.346	1.372	0.266	0.514	1.305	0.360	0.403	1.433
Junior	−0.013	0.969	0.987	0.076	0.848	1.079	0.137	0.749	1.147
Bachelor and above	0.523	0.201	1.687	0.115	0.818	1.121	0.115	0.831	1.122
*Occupation (Reference: Government)*									
Company	−1.705**	0.001	0.182	−1.557^**^	0.005	0.211	−1.514**	0.008	0.220
Farmer	−2.485***	<0.001	0.083	−1.821**	0.002	0.162	−2.045**	0.001	0.129
Flexible	−2.153***	<0.001	0.116	−1.847**	0.001	0.158	−1.953**	0.001	0.142
Other	−2.249***	<0.001	0.106	−1.914**	0.001	0.148	−1.897**	0.001	0.150
*Emphasizing health management (Reference: No)*									
Yes	0.500*	0.013	1.649	0.579*	0.015	1.784	0.646*	0.011	1.909
Enabling resources									
*Family size (Reference: 1-2)*									
3–4				−0.465	0.257	0.628	−0.531	0.211	0.588
5–6				−0.828	0.055	0.437	−0.837	0.064	0.433
7 ~ 8				−0.721	0.223	0.486	−0.706	0.251	0.493
≥9				−0.543	0.460	0.581	−0.311	0.682	0.733
*Annual household income (Reference: ≤60,000)*									
60,000–100,000				0.601*	0.036	1.823	0.827**	0.008	2.288
100,000–150,000				1.192**	0.002	3.294	1.714***	<0.001	5.553
150,000–200,000				1.125	0.055	3.081	1.813**	0.005	6.130
>200,000				0.003	0.997	1.003	0.405	0.651	1.500
*The degree of understanding of supplementary medical insurance (Reference: very unfamiliar)*									
Slight unfamiliar				−0.716	0.055	0.489	−0.832*	0.036	0.435
Moderately familiar				−0.522	0.160	0.594	−0.545	0.166	0.580
Quite familiar				−1.726***	<0.001	0.178	−1.952***	<0.001	0.142
Very familiar				−1.630**	0.004	0.196	−1.704**	0.005	0.182
*Has paid supplementary medical insurance (Reference: No)*									
Yes				1.925***	<0.001	6.855	2.007***	<0.001	7.437
*The degree of influence of insurance prices (Reference: very impact)*									
Quite impact				0.006**	0.986	1.006	−0.175	0.644	0.839
Moderate impact				0.271	0.424	1.312	0.152	0.679	1.165
Slight impact				1.208**	0.003	3.347	1.061**	0.015	2.889
No impact				1.461*	0.018	4.312	1.634**	0.013	5.126
*The degree of influence of payment convenience (Reference: very impact)*									
Quite impact				−1.145*	0.022	0.318	−1.066*	0.042	0.344
Moderate impact				−1.058*	0.037	0.347	−0.849	0.109	0.428
Slight impact				−0.740	0.194	0.477	−0.527	0.373	0.590
No impact				−1.108	0.119	0.330	−1.212	0.104	0.298
Demand factors									
*Health condition in the past year (Reference: very good)*									
Better							−0.184	0.555	0.832
Generally							0.572	0.206	1.773
Poor							1.268	0.136	3.552
Very poor							2.033	0.355	7.639
*The number of medical visits in the past year (Reference: 0)*									
1–3							0.135	0.679	1.144
4–7							−0.119	0.792	0.888
8–12							−0.124	0.890	0.883
≥13							−0.433	0.816	0.648
*Family medical expenses in the past year (Reference: ≤3,000)*									
3,000–8,000							−0.228	0.466	0.796
8,000–15,000							−1.291**	0.003	0.275
15,000–30,000							0.043	0.948	1.044
>30,000							−0.599	0.433	0.550
*The compensation of basic medical insurance meets the demand (Reference: No)*									
Yes							−0.819**	0.005	0.441
Model fitting results									
Cox & Snell R^2^	0.150			0.316			0.348		
Nagelkerke R^2^	0.202			0.426			0.470		
Log-likelihood estimation value	656.467			536.574			510.048		

“*, **, and ***” respectively represent *P* < 0.05, *p* < 0.01, and *p* < 0.001.

Across the three regression models, the three major categories of factors in the Andersen framework each showed significant associations, to varying degrees, with willingness to pay for supplementary medical insurance among rural residents in ethnic minority areas. In terms of model fit, as additional blocks of variables were introduced, the Cox &Snell R^2^ squared values increased from 0.150 to 0.316 and then to 0.348, while the Nagelkerke R^2^ squared values rose from 0.202 to 0.426 and then to 0.470. Meanwhile, the log likelihood value decreased from 656.467 to 510.048. Taken together, these results indicate a progressive improvement in overall model fit and a corresponding increase in the models’ explanatory power for rural residents’ willingness to pay for supplementary medical insurance.

Specifically, among tendency characteristics, sex, age, marital status, and education level were not significantly associated with willingness to pay in any of the three models. Occupation showed a stable and significant association with willingness to pay across all models. The association between attention to health management and willingness to pay became stronger as additional blocks of variables were added. After enabling resources were introduced, family size remained not significant. In contrast, annual household income, understanding of supplementary medical insurance, prior purchase experience, insurance prices, and purchasing convenience were significantly associated with willingness to pay in Models 2 and 3, with annual household income, prior purchase experience, and insurance prices showing comparatively stronger associations. After demand factors were further included, self rated health status and the number of healthcare visits in the past year were not significantly associated with willingness to pay. However, family medical expenses and whether reimbursement from basic medical insurance met residents’ healthcare needs were significantly associated with willingness to pay for supplementary medical insurance. Overall, adding enabling resources led to the largest improvement in the model’s explanatory power, indicating that enabling resources in the Andersen model play a particularly important role in rural residents’ willingness to pay. The inclusion of demand factors further refined the model structure on this basis. Accordingly, Model 3 showed the strongest overall explanatory power and is therefore the main focus of the following discussion and analysis.

In addition, the survey wave variable was not statistically significant in any of the three models. This suggests that after controlling for tendency characteristics, enabling resources, and demand factors, survey timing did not have a significant association with willingness to pay for supplementary medical insurance. Therefore, pooling the two survey waves for analysis is statistically justifiable. Moreover, based on a review of relevant materials and consultations with local staff, no major changes occurred in Tongdao County between the two survey periods with respect to key policies on supplementary medical insurance or insurance prices.

### 3.3 Multiple correspondence analysis of willingness to pay and influencing factors

**Figure 2:**
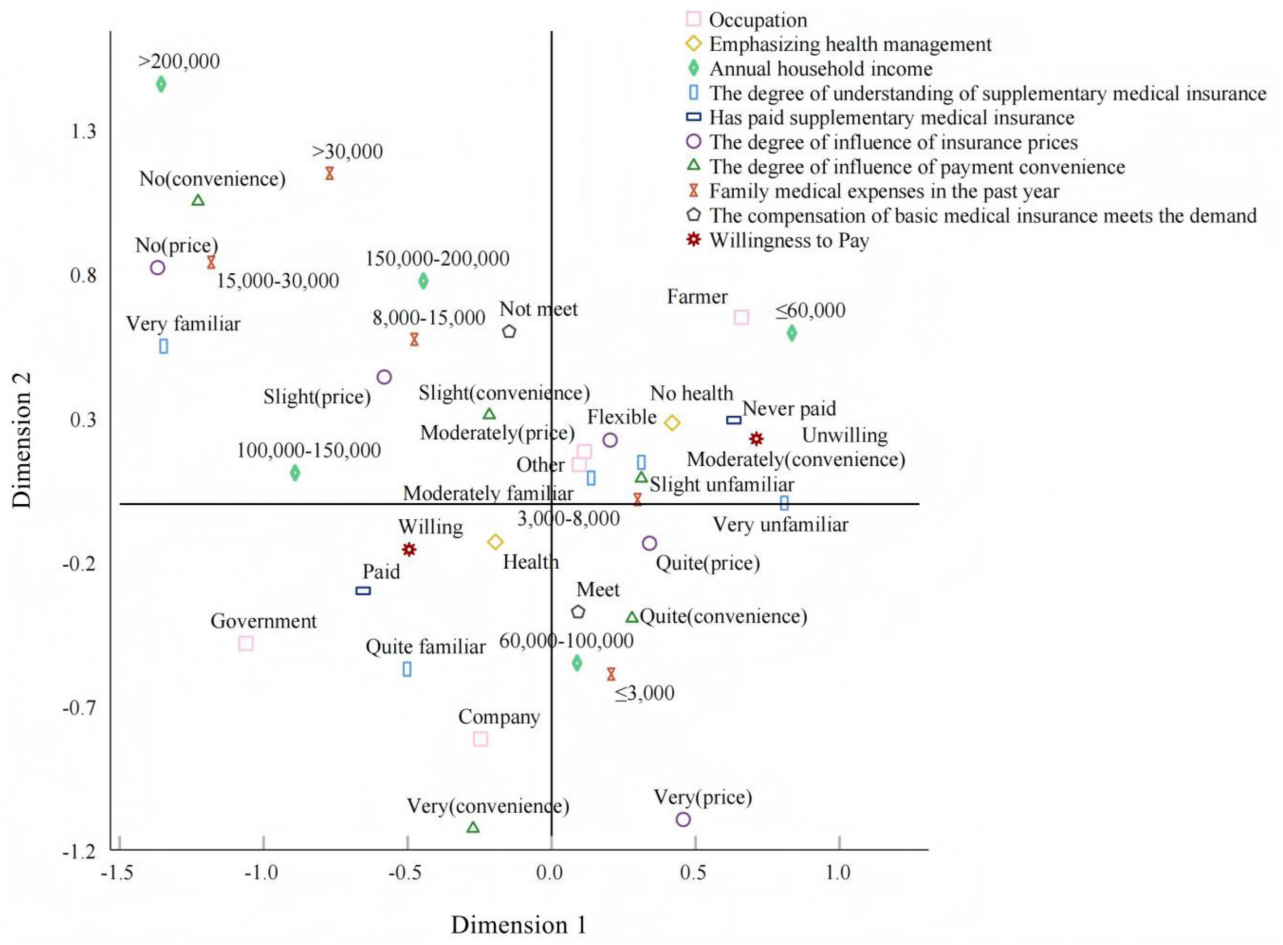
Multiple correspondence analysis of willingness to pay and influencing factors.

To further explore the underlying association structure and potential patterns among the variables influencing rural residents’ willingness to pay for supplementary medical insurance (28), we conducted multiple correspondence analysis using willingness to pay and all variables that were significant in logistic regression Model 3, as shown in Figure 2. The results showed that unwillingness to pay clustered spatially with low annual household income, being flexibly employed or a farmer, not paying attention to health management, having no prior purchase experience of supplementary medical insurance and low awareness of it, being more sensitive to insurance prices, and perceiving purchasing convenience as having only a moderate influence. This suggests that unwillingness to pay is not determined by a single economic factor. Simply increasing residents’ income is unlikely to substantially raise willingness to pay in this population. Improving health management and insurance awareness, and reducing perceived payment barriers, are also important. Willingness to pay was more closely associated with higher annual household income, employment in government agencies or enterprises, prior purchase experience, greater familiarity with supplementary medical insurance, and stronger attention to health management. This pattern suggests that higher willingness to pay is often linked to previous insurance participation, better awareness and understanding, and a more stable occupational background. Accumulated experience and knowledge may further increase willingness to pay by reducing price sensitivity and shaping perceptions of risk.

## 4 Discussion

### 4.1 Enabling resources play a key role in residents’ willingness to pay

Enabling resources played a key role in rural residents’ willingness to pay for supplementary medical insurance. First, annual household income had a significant association with willingness to pay, consistent with the findings of Thsehla et al. (25). Compared with residents whose annual household income was below 60,000 yuan, those with an income of 60,000–100,000 yuan (OR = 2.288), 100,000–150,000 yuan (OR = 5.553), and 150,000–200,000 yuan (OR = 6.130) were significantly more willing to pay, with stronger willingness as income increased. By contrast, income above 200,000 yuan was not statistically significant. This indicates that, in rural areas of Tongdao County, middle income households show stronger willingness to pay than low income households. Against the backdrop of county level economic catch up, most rural households in Tongdao County are currently in a transition stage, consolidating poverty alleviation gains while working toward higher incomes (29). Although middle income households have some ability to pay, they are highly sensitive to the risk of catastrophic medical expenditure caused by major illness. As a result, they are more inclined to purchase insurance to mitigate medical risks and reduce the likelihood of falling back into poverty due to illness (35). Second, prior purchase experience was the strongest positive factor associated with willingness to pay in the regression results (OR = 7.437). Residents with prior purchase experience had higher willingness to pay than those without such experience (OR = 2.007), which is consistent with the findings of Jung and Ha (20). When individuals lack prior experience with supplementary medical insurance and few people in their social environment are familiar with or pay for such products, their understanding of this form of insurance is often limited, and they may even develop misconceptions. These misunderstandings can reduce their willingness to pay. This finding suggests that the promotion of supplementary medical insurance in rural areas remains inadequate and that residents still have limited awareness of its importance. It also indicates that prior insurance experiences can significantly strengthen residents’ trust in and willingness to pay for supplementary medical insurance. However, compared with residents who reported being very unfamiliar with supplementary medical insurance policies, those who considered themselves very familiar (OR = 0.182) or relatively familiar (OR = 0.142) were less willing to pay. This finding differs from the results reported by Geng et al. (19). This finding points to a deeper challenge in health insurance outreach in Tongdao County. According to the Tongdao County Medical Security Bureau’s 2024 annual work report, the bureau noted that current publicity efforts have limited effectiveness and that both content and delivery lack innovation and appeal (30). Residents’ self reported familiarity may therefore reflect negative hearsay, such as claims that reimbursement is difficult or not cost effective, rather than a clear understanding of policy terms and government support measures. This can create a trust barrier to supplementary medical insurance. In addition, compared with residents who reported that insurance prices had a very large influence, those who reported that insurance prices had a slight influence (OR = 2.889) or no influence (OR = 5.126) were more willing to pay. This suggests that insurance prices remain an important practical constraint on willingness to pay in rural areas. Similarly, purchasing convenience also influenced residents’ willingness to pay. Overall, enabling resources include not only households’ objective economic conditions but also insurance participation experience, awareness and understanding, and perceived conditions related to policy implementation. Together, these factors shape rural residents’ willingness to pay for supplementary medical insurance.

### 4.2 Tendency characteristics have a substantial influence on willingness to pay

Tendency characteristics reflect differences in individuals’ attitudes, values, and social roles. They exert a substantial influence on individuals’ willingness to pay. The results of this study indicate that emphasizing health management has a significant positive influence on residents’ willingness to pay (OR = 1.909). This aligns with the conventional pathway whereby individuals, after perceiving health risks, proactively purchase insurance to mitigate those risks. Residents who place greater emphasis on routine health management are more likely to recognize the value of supplementary medical insurance in sharing the burden of high medical expenses, thereby exhibiting a higher willingness to pay (25). This further indicates that health management is a significant driver of willingness to pay among rural residents. Enhancing health promotion and education could indirectly increase residents’ acceptance of supplementary medical insurance. Furthermore, occupation also significantly influences willingness to pay (23). Compared with residents employed in Party, government, or public institutions, those working in enterprises, engaged in farming, in flexible employment, and in other occupations all exhibited significantly lower willingness to pay (OR = 0.22, OR = 0.129, OR = 0.142, OR = 0.15). Occupational status not only affects income but is also associated with residents’ level of understanding of supplementary medical insurance and the accessibility of payment channels. Residents with stable positions and benefits often have heightened awareness and acceptance of supplementary medical insurance due to relatively comprehensive supplementary coverage provided through their workplaces. In contrast, individuals without formal employment or in flexible employment may exhibit lower willingness to pay due to income instability, the psychological burden associated with premium payments, and information asymmetry. In addition, sex, age, and education level were not statistically significant in the regression models. This differs from the findings reported by Teng and Li (21). A possible explanation is that their effects were confounded by more proximate variables included in the model. For example, the influence of education level may operate through occupation and annual household income.

### 4.3 Demand factors have a moderate influence on willingness to pay

In this study, demand factors reflect the actual degree of residents’ need for supplementary medical insurance. Among these factors, family medical expenses was significantly associated with willingness to pay (14). Compared with residents whose family medical expenses in the past year was 3,000 yuan or less, those with expenditure of 8,000–15,000 yuan were 1.291 times less willing to pay (OR = 0.275). This finding contrasts with the conventional expectation that higher medical expenditures would lead to a greater perception of financial burden and, hence, a stronger inclination to purchase insurance. In light of the local context in Tongdao County, the county has effectively contained patients’ out of pocket medical burden by strictly implementing centralized volume based procurement for medicines and medical consumables and by deepening reform of the DIP payment method. These measures have reduced the average prices of medicines and consumables by more than 50 percent, and both inpatient reimbursement indicators and the share of expenses outside the reimbursement catalogue have fallen to less than half of the required threshold. In addition, an early warning mechanism for high medical expenses, together with timely medical assistance, has provided a risk buffer for households with moderate levels of medical expenditure (30). Therefore, the relatively lower willingness to pay among these residents suggests that the existing system has, to some extent, alleviated their immediate risk of catastrophic medical expenditure, thereby reducing their willingness to pay for supplementary medical insurance. Similarly, residents who believed that the compensation of basic medical insurance adequately met their needs exhibited a reduced willingness to pay for supplementary insurance compared with those who believed their needs were not met (OR = 0.441). This is consistent with the findings of Pan et al. (31) and aligns with the substitution effect in quantitative economics. When the availability of, and satisfaction with, basic coverage improves, the marginal demand for additional coverage tends to decline. This suggests that as Tongdao County continues to consolidate coverage under basic medical insurance and gradually improves outpatient and inpatient reimbursement policies, rural residents’ institutional trust in basic coverage has strengthened. It also implies that, to stimulate effective demand, supplementary medical insurance must adopt a clear differentiated positioning, rather than competing with basic medical insurance through simple homogenization.

### 4.4 Disconnect between willingness to pay and actual behavior among residents in Tongdao County

Both multiple correspondence analysis and logistic regression analysis indicated a close and significant association between payment experience and willingness to pay. According to the survey data, 58.98% of rural residents in Tongdao County were willing to pay for supplementary medical insurance, while only 49.18% had actually pay for such insurance without considering policy continuity. This indicates that residents’ willingness to pay has not been fully translated into actual payment behavior, revealing a clear “willingness-behavior” disconnect. The gap between willingness and behavior can be attributed to several reasons. First, relevant policies and systems are incomplete. At present, supplementary medical insurance policies in Tongdao County mainly comprise medical insurance benefits for disadvantaged groups and specific subsidies for civil servants and employees. Formal policies and measures for other groups have not yet been introduced, leaving other residents without direct access channels and resulting in insufficient understanding of the functions and value of supplementary medical insurance. Second, product and service supply is inadequate. The investigation revealed that only three professional commercial insurance companies currently operate in Tongdao County, and their business activities in rural areas, as well as their promotional outreach efforts, are limited. Insurance companies lack strong motivation to enhance rural residents’ willingness to pay. Third, the occupational structure of rural residents is predominantly characterized by farming and flexible employment, with low levels of organization, which restricts collective mobilization and overall improvements in payment rates. The operation of supplementary medical insurance relies on the “law of large numbers.” The greater the number of paying participants, the more significant the risk-pooling effect, which ensures the stability and sustainability of the insurance products. The greater the number of paying participants, the more pronounced the risk-pooling effect, ensuring the stability and sustainability of the insurance products. A low payment rate not only undermines the construction of the risk pool but may also weaken the products’ long-term development capacity. Therefore, effectively converting residents’ potential willingness to pay into actual payment behavior is particularly crucial in the promotion of supplementary medical insurance in Tongdao County.

## 5 Recommendations

### 5.1 Strengthening government leadership and mechanism construction

Local governments should exercise their autonomous functions in system design, policy planning, and awareness promotion, guided by higher-level medical insurance policies for ethnic minority regions (32). First, the county government should take the lead in launching an inclusive supplementary medical insurance product at the county level. Tongdao County could draw on the “Hunan Huiminbao model” by partnering with commercial insurers to design an inclusive product for all residents enrolled in basic medical insurance across the county, with low premiums, broad eligibility, and strong protection against major illnesses. Given that purchase experience is the most powerful factor driving payment willingness, a combination of one-click insurance enrollment through online platforms and collection at township medical insurance service outlets can be adopted to maximize the reduction of the initial insurance enrollment threshold for residents, thereby increasing their experience in purchasing supplementary medical insurance. Second, targeted enrollment subsidies should be provided to middle income households. Research shows that the middle-income group with an annual household income of 100,000 to 200,000 yuan has the strongest willingness to pay but is also a vulnerable group under the impact of major illness risks. In the short term, part of the funds from rural revitalization or medical and health special funds can be allocated (33) to provide a fixed direct subsidy for the first insurance coverage for such families, such as a 30% reduction in insurance premiums. Third, a transparent information and education platform should be established to address the cognition paradox whereby residents who consider themselves familiar with insurance show lower willingness to pay. The county medical security bureau should upgrade its current outreach approach by creating an official Tongdao County Multilevel Medical Security information column and regularly publishing verified local claims cases, comparison lists highlighting differences in coverage, and cost estimation tools. Finally, medical security authorities and operating agencies should strengthen operational guidance and support for supplementary medical insurance in ethnic minority areas. Regulatory authorities should ensure that the medical insurance market operates transparently and in a well regulated manner through measures such as information disclosure and accessible complaint mechanisms.

### 5.2 Optimizing insurance products and service supply

As the product provider, insurers should offer coverage plans that genuinely align with local residents’ risk profiles and ability to pay. First, insurers should develop differentiated products that complement basic medical insurance (34). Given that believing that basic medical insurance payouts can meet the needs would reduce residents’ willingness to pay for supplementary medical insurance, insurance institutions must conduct precise analysis of the coverage gap, clearly focusing on areas such as high-cost drug expenses outside the basic medical insurance coverage, compensation for income loss after major diseases, and better medical service options. These differentiating features should be clearly specified in both policy contracts and promotional materials to avoid homogenized competition. Insurers could also take into account the county’s common disease profile and the diagnostic and treatment characteristics of ethnic medicine in Tongdao County, and explore benefit designs with local features. Concurrently, emphasis should be placed on improving residents’ payment experience. Measures such as optimizing the claims process, shortening settlement cycles, and enhancing service transparency can continuously build residents’ trust and satisfaction (27). Incentives for initial payments, such as health check-up vouchers or medication discounts, could be offered to encourage first-time uptake and to continuously drive the conversion of willingness to pay into actual payment behavior. Furthermore, insurance companies should explore collaborations with primary healthcare institutions and village collectives. Integrating supplementary medical insurance with services such as health management and chronic disease follow-ups can enhance product stickiness and sustainability.

### 5.3 Improving health literacy and risk awareness

Residents’ perceptions of health risks and their understanding of supplementary medical insurance significantly influence both their willingness to pay and their actual payment behavior. First, residents should proactively use official channels to improve their insurance awareness and ability to assess information, rather than passively receiving messages. When considering the purchase of supplementary medical insurance, they should learn to use official comparison tools and focus on key policy terms, including the scope of coverage, deductibles, reimbursement rates, and exclusions. This can support product comparisons and informed, rational decision making, helping to avoid rejection due to misunderstandings or purchases made without adequate information. Second, households should strengthen health management and awareness of medium to long term financial risk planning. Research has confirmed that residents who attach importance to health management have a higher willingness to pay. While enjoying the basic medical insurance and medical assistance as the bottom-line protection, residents should, based on their family’s health conditions and economic capacity, actively assess the potential huge risks of medical expenditures, and view supplementary medical insurance as an important tool for hedging family financial risks and make long-term plans accordingly. Finally, communities should foster positive neighborhood interactions and experience sharing. Residents who have enrolled in supplementary medical insurance and benefited from it should be encouraged to share real and specific experiences within village collectives. Such first hand accounts from trusted acquaintances can gradually counter negative stereotypes about insurance, help cultivate a rational approach to risk and proactive planning for protection, and thereby promote the uptake of supplementary medical insurance from the demand side.

## 6 Limitations of the study

This study has several limitations that should be considered when interpreting the findings. Firstly, the sampling strategy of this study is not strictly a probability sampling. The data were collected through intercept surveys in township market areas and online questionnaires distributed via social media. This mixed method is closer to convenience sampling and self-selection sampling rather than simple random sampling, which may limit the representativeness of the sample. Secondly, the data were collected at two different time points. Although the survey period was controlled in the multiple regression model and no statistically significant impact was shown, combining the observation results from the two time points still might introduce unobserved temporal heterogeneity. Furthermore, the explanatory power of the regression model is relatively limited. The Nagelkerke R^2^ value of the final model is 0.47, indicating that approximately 47% of the differences in willingness to pay are explained by the included variables. Although this level of fit is acceptable for cross-sectional behavior research, it also means that a considerable portion of the differences remain unexplained and the current model fails to capture other relevant factors. Finally, the selection of explanatory variables is limited by data availability and measurement design. The model mainly focuses on individual-level characteristics and does not include variables at the community or institutional level, such as local medical supply or insurance service density, which may also affect willingness to pay.

## 7 Conclusion

Based on the Andersen model research framework, this study designed a questionnaire and conducted a systematic analysis of willingness to pay for supplementary medical insurance and its influencing factors among residents in the rural areas of Tongdao Dong Autonomous County, Hunan Province. The logistic regression model results indicated that all three categories of factors within the Andersen model framework significantly influenced willingness to pay among rural residents in Tongdao County. Specifically, income level and prior insurance experience among the enabling resources directly determine residents’ ability and resource conditions for payment. Health management awareness and occupational status among the tendency characteristics influence residents’ attitudes and perceptions toward insurance. The demand factors are reflected in the roles of medical expenditures and satisfaction with basic medical insurance in shaping the perceived value of supplementary medical insurance. Multiple correspondence analysis revealed close associations between rural residents’ willingness to pay and their occupation, income, and purchase experience, indicating the significant roles of economic status and occupation in shaping payment willingness. Overall, although rural residents in Tongdao County exhibit a foundational willingness to pay, this has not been fully translated into actual and effective payment behavior, and the payment rate still requires further improvement. Therefore, the development of supplementary medical insurance for rural residents in Tongdao County requires collaborative efforts from multiple stakeholders to foster a stable and sustainable development pattern. This will provide solid support for safeguarding the health rights of residents in this region and for reducing the risks of falling into poverty due to illness or returning to poverty because of illness.

## Data Availability

The original contributions presented in the study are included in the article/supplementary material, further inquiries can be directed to the corresponding author.
