# Supplementary material for: Willingness to pay for supplementary medical insurance and its influencing factors among rural residents in ethnic minority areas
Source: Front Public Health. 2026 Mar 4;14:1733751. doi: 10.3389/fpubh.2026.1733751 (PMC12996166; doi:10.3389/fpubh.2026.1733751)
Supplement: Supplementary file 1 [file Data_Sheet_1.pdf]

Xuzhou Medical University
Recommendation Form for Ethical Review of Scientific Research Projects（Revision1th）

RecommendedProject No.:XZHMU-25Z1002   Date: May 21, 2025

**Project title:** Analysis of the Willingness to Participate in Supplementary Medical Insurance and Influencing Factors among Residents in Rural Areas of Ethnic Minority Regions
**Project leader:** Shen Liang   **Title:** Associate Professor<br>**Institution:** School of Management, Xuzhou Medical University
**Project contact:** Shen Liang   **Phone:** 18361216705<br>**Email:** liang_shen@xzhmu.edu.cn
**Research category:**<br>☑1.Philosophy and Social Sciences<br>☐2.Involving Human Subjects (Including experiments involving human tissues or cells)   ☐3. Other________
**Project category to be applied for:** Xuzhou Social Science Fund Project
**Project funding source:** ☑Government ☐Foundation ☐Company ☐International organization ☐Other____
**Research start and end time:** May 1, 2025 - December 31, 2025
**Materials to be submitted for review:** ☐Project plan ☐Project application ☐Research paper<br>☑Other__Research abstract__

Research contents and research design summary

Research content 1: Measurement and Current Status of Minority Rural Residents' Willingness to Participate in Supplementary Medical Insurance

Drawing upon the Andersen Behavioral Model of Health Service Utilization, this study establishes an analytical framework to examine factors influencing residents' willingness to participate in supplementary medical insurance. Integrating risk perception and perceived value theories, a self-administered questionnaire will be developed to assess the willingness of rural residents in Tongdao Dong Autonomous County, Hunan Province. By investigating socioeconomic characteristics, health cognition, risk perception, and attitudes toward insurance participation, the study seeks to capture the overall level and structural features of participation willingness, providing an empirical foundation for mechanism analysis.

Research content 2: Influencing Factors of Minority Rural Residents' Willingness to Participate and the Mechanism of the Andersen Model

Employing univariate and multivariate analyses, this section identifies major determinants of participation willingness and investigates the mechanisms operating within the Andersen model framework. It explores how predisposing characteristics (health awareness, risk perception, etc.), enabling resources (economic capacity, accessibility of insurance, etc.), and need factors (health status, medical expenditure, etc.) jointly shape participation willingness through direct and indirect pathways. The analysis further examines the underlying causes of these relationships and compares them with findings from previous studies to refine the theoretical model.

Research Content 3: Multiple Correspondence Analysis of Key Factors Influencing Residents' Willingness to Participate

Based on survey data and prior analytical results, this section applies multiple correspondence analysis (MCA) to identify patterns of association among key variables affecting participation willingness. By constructing a multidimensional interaction model, the study examines how demographic characteristics, economic capacity, insurance cognition, and medical burden are distributed across different population subgroups. The findings will provide support for targeted interventions and differentiated policy design.

Research Content 4: Comparative Study of Participation Willingness and Actual

Enrollment Behavior

From the perspective of behavioral transformation, this section compares residents' stated willingness to participate with their actual enrollment behavior to explore the mechanisms underlying the discrepancy between intention and action. A "willingness – behavior" relational model will be constructed to analyze how economic constraints, policy awareness, product design, and institutional trust affect the transformation process, and to uncover the deeper social and structural factors that hinder behavioral realization.

Research Content 5: Policy Pathways to Promote the Transformation of Participation Willingness and System Optimization

Building on the preceding analyses, this section proposes actionable policy pathways to promote the transformation of participation willingness into actual enrollment among minority rural residents. It emphasizes the coordination mechanisms among government agencies, insurance providers, grassroots organizations, and local communities. The study explores policy instruments such as differentiated premium design, targeted information campaigns, and product and service improvements, aiming to enhance the inclusiveness and sustainability of supplementary medical insurance in rural minority regions.

Research Design

This study focuses on minority rural populations and systematically examines the formation and transformation mechanisms of their willingness to participate in supplementary medical insurance. Anchored in the Andersen Behavioral Model and enriched by risk perception and perceived value perspectives, the study will employ a structured questionnaire encompassing socioeconomic attributes, health status, insurance cognition, and risk attitudes. Field surveys and data analyses-including descriptive statistics, chi-square tests, logistic regression, and multiple correspondence analysis will be conducted to identify influencing factors and interpret their underlying mechanisms. The study aims to clarify the current status and key determinants of participation willingness, and to develop evidence-based policy recommendations that facilitate the conversion of willingness into actual participation. Ultimately, it seeks to contribute theoretical and empirical insights for optimizing the multi-tier medical security system and advancing equitable health insurance policies in rural minority areas.

Review results of the school ethics committee

☑Agree ☐Agree with necessary amendments ☐Re-examine with necessary amendments

☐Disagree ☐Terminate or suspend the trial

Official seal:

Date: May, 21, 2025
